# Strategies for Pre-Emptive Mid-Air Collision Avoidance in Budgerigars

**DOI:** 10.1371/journal.pone.0162435

**Published:** 2016-09-28

**Authors:** Ingo Schiffner, Tristan Perez, Mandyam V. Srinivasan

**Affiliations:** 1 Queensland Brain Institute, University of Queensland, St Lucia, QLD, Australia; 2 Elec. Eng. and Comp. Sc., Queensland University of Technology, Brisbane, QLD, Australia; 3 School of Information Technology and Electrical Engineering, University of Queensland, St Lucia, QLD, Australia; University of Sussex, UNITED KINGDOM

## Abstract

We have investigated how birds avoid mid-air collisions during head-on encounters. Trajectories of birds flying towards each other in a tunnel were recorded using high speed video cameras. Analysis and modelling of the data suggest two simple strategies for collision avoidance: (a) each bird veers to its right and (b) each bird changes its altitude relative to the other bird according to a preset preference. Both strategies suggest simple rules by which collisions can be avoided in head-on encounters by two agents, be they animals or machines. The findings are potentially applicable to the design of guidance algorithms for automated collision avoidance on aircraft.

## Introduction

With the ever increasing density of air-traffic and operations pushing for the use of unmanned aircraft, there is a pressing need for developing robust automatic sense and avoid solutions for both manned and unmanned aircraft [1, 2]. In order to assist with the development of these solutions, it is useful to investigate behaviours in animals that could potentially be applicable to aircraft. Birds have taken to the sky 150 million years ago [3] and insects 350 million years ago [4], and are likely to have evolved robust solutions for collision avoidance. While insects are relatively low in mass and possess an exoskeleton that provides a layer of protection, a bird is heavier, flies at faster speeds, and its structure is more fragile. Birds, therefore, must have been under strong evolutionary pressure to establish basic rules and strategies to minimize the risk of collision in advance. Surprisingly, very little is known about collision avoidance in birds. Past studies have focused on obstacle avoidance or passage through narrow apertures [5, 6] or avoidance of mates in a flock [7], but no studies have looked specifically at what happens when two birds fly towards each other. The purpose of the present study is to evaluate birds’ general abilities and strategies for collision avoidance. In a series of experiments, we released pairs of birds from opposite sides of a flight tunnel to look for general strategies that they might use to minimize the risk of collisions. We pose the potential behaviours of interest as propositions or hypotheses in a Bayesian framework to compute the predictive probability of these propositions [8] to arrive at robust conclusions about the collision-avoidance strategies used by the birds.

## Materials and Methods

### Ethics Statement

All the experiments in this study have been conducted in accordance with the Australian Law on the Protection and Welfare of Laboratory Animals and with the approval of the Animal Experimentation Ethics Committee of the University of Queensland, Brisbane, Australia.

### Experimental Setup

**Fig 1:**
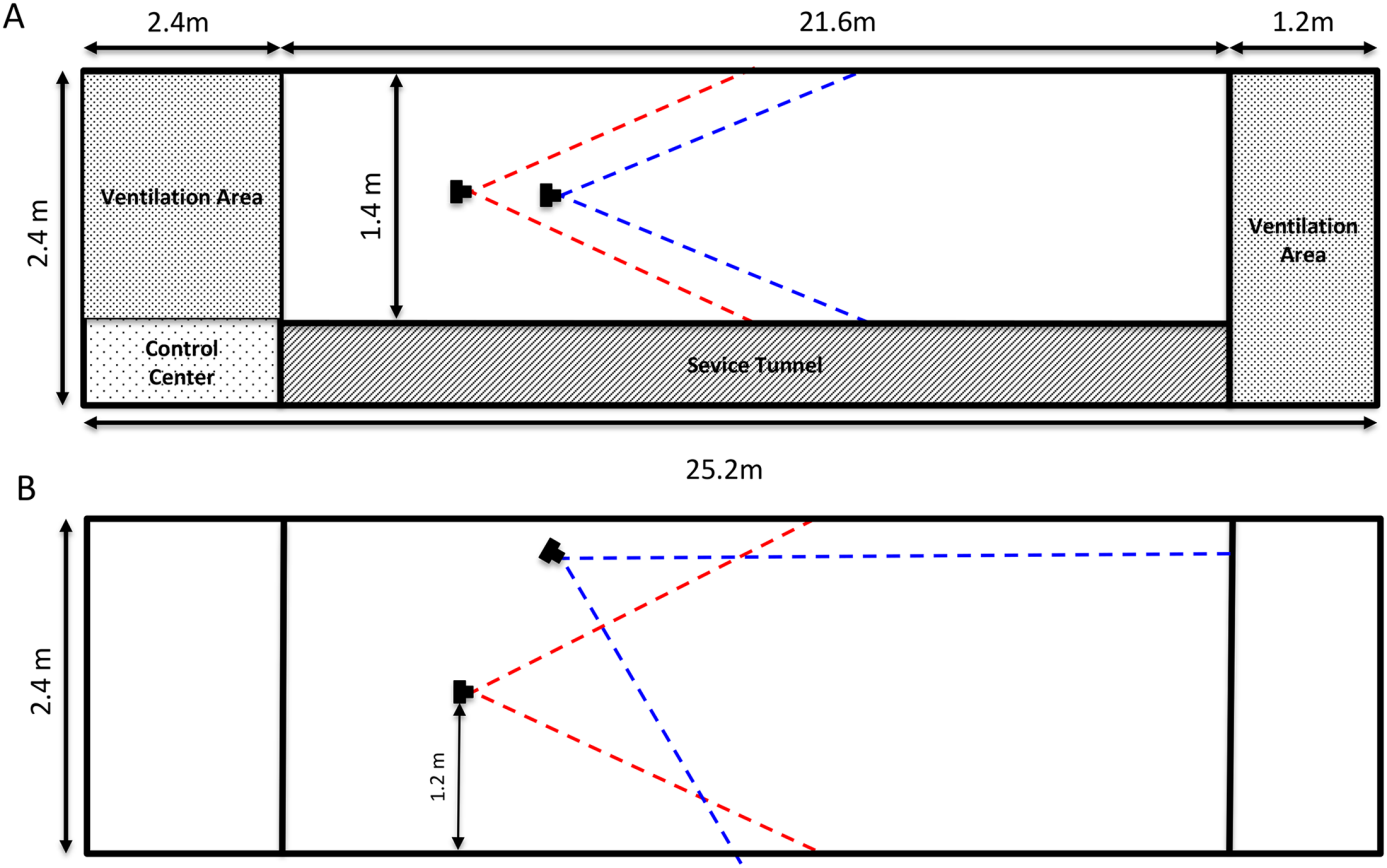
Side elevation (A) and plan (B) view of flight tunnel. Red and blue dashed lines indicate the fields of view of the respective cameras. Note: figure is not to scale.

The experiments were conducted in a purpose-built bird-flight tunnel (height: 2.40m, width: 1.40m length 21.6m; See Fig 1).

The walls of the tunnel were white, the floor was a light grey, The ceiling was white, and carried florescent lights that provided flicker-free (4000 Hz) illumination. To ensure that the birds could see each other clearly, we avoided the use of any potentially distracting visual patterns on the floor or the walls. Under these conditions, the birds were found to fly and orient normally, without any apparent difficulty.

A group of ten male budgerigars—*Melopsittacus undulatus*—were trained to fly along the tunnel. All flights were recorded using two synchronized high-speed video cameras (Motion Pro) at 120 frames per second. One camera was positioned with its optical axis aligned with the longitudinal axis of the tunnel, and the other camera was mounted on the ceiling with its optical axis tilted downwards at an angle.

Two birds were released from the opposite ends of the tunnel, at an initial separation of either 5 m or 10 m from each other. Two distances were used in order to examine whether there were differences in behavior based on the time the birds had to react to the presence of the other bird. Prior to the experiments, the birds were flown only singly in the tunnel and had therefore never previously experienced the situation of encountering another bird in the tunnel. However, the birds were familiar with the tunnel. Each pair was chosen at random and was tested up to 10 times, for both initial distances. The starting positions were interchanged after each trial. While the birds were generally willing to fly against each other, some pairs of birds proved to be difficult, which is the reason why, fewer than 10 flights were recorded in some cases. Over the course of 4 days, we tested a total of 7 pairs, consisting of 10 individual birds, and recorded a total of 102 flights. No collisions were observed in the entire study.

### Analysis of Behaviour

After recording, the video from each camera view was evaluated by visual inspection, looking specifically at the time window when the birds passed each other. From this, we scored and evaluated independently the following behaviours defined with respect to the bird that commences the flight on the left-hand side of the tunnel as depicted in Fig 1 (we term this the reference bird):

1. Minimum relative distance was scored as specified in Table 1;
2. Tendency of the reference bird to veer to the right while passing the other bird;
3. Tendency of the reference bird to pass either above or below the other bird;

**Table 1:** Behaviours and proximity scores.

Behaviour	Proximity Score
Very close encounter (near miss)	1
Close encounter	2
Medium separation encounter	3
Large separation encounter	4

The scores are subjective and not related to the dimensions of the birds or their wing span.

In some of the experiments, it was not possible to evaluate behaviours b (tendency of the reference bird to veer to the right while passing the other bird) and c (tendency of the reference bird to pass either above or below the other bird) reliably, because of the birds’ proximity. In some cases, birds flew at the same height, avoiding collisions by moving to the side. In other cases, birds flew through the center of the tunnel, but at different heights. In such experiments, observers were asked to score only the behaviour that could be assessed unambiguously. To avoid observer bias, the data was scored independently by two observers. Observer 1 did not know how observer 2 scored a certain behavior, and vice versa. The two observers provided scores that were strongly consistent. All scored behaviours were tested and compared using a two-sample t-test to test for differences in the behaviour between the releases from 10m and 5m.

### Testing Propositions About Behaviours

In order to gain knowledge about the behaviours observed during impending collisions, a natural approach is to pose behaviours as propositions (or hypotheses) *H*_*i*_–something that can either be true or false–and form a judgement about their likely truth or falsity by computing their probabilities conditional on all the evidence at hand [8]. That is, we can use these probabilities to describe our state of knowledge about a proposition based on our current information.

In particular, we consider the following propositions related to behaviours for each bird (*j* = 1, 2, …, *N*_*B*_) in our experiments:

- $H_{1}^{jk}$ = {the reference bird *j* has a preference to veer towards the right when encountering bird *k*};
- $H_{2}^{jk}$ = {the reference bird *j* has a preference to increase or decrease its altitude when encountering bird *k*}.

Note that the choice of ‘right’ in proposition $H_{1}^{jk}$ is arbitrary; it could have been the other way around, and this does not affect the results or conclusions. If we consider the data *D* and the background information *B*_*ijk*_ then we can compute the predictive probabilities *P*(*H*_*i*_|*D*, *B*_*ijk*_), *i* = 1, 2. *B*_*ijk*_ specifies that we are considering the hypothesis *i* = 1, 2 for the *jk*-bird pair. Our Bayesian approach to the analysis of binary choice data, summarised briefly here and described in detail in S1 Supplementary Methods, is a powerful way of extracting statistically meaningful information from a restricted data set [8–10].

## Results and Discussion

In an initial analysis, we were able to exclude any effects of release distance (See Table 2). None of the behaviors scored in the study were significantly affected by distance (all scored behaviors; two-tailed-t-test *p* > 0.05). Specifically, the proximity score was independent of release distance, with birds released at 10m displaying an average Proximity score of 3.12±0.65 and birds released at 5m an average Proximity score of 3.3±0.74 (Two Sample T-test *p* = 0.41). This suggests that the time the birds had when they were released at a separation of 5 m was still sufficient for detection and evasive action. Assuming that both birds flew at a speed of 6 ms^−1^, on average, based on data from preliminary tracking analysis, this means that birds had roughly 0.42s to detect each other and initiate avoidance maneuvers. This is consistent with findings in obstacle avoidance tasks, where changes in behaviour can be observed when the bird is already relatively close to the object, usually in the range of approximately 0.25s (1.5m) [5, 6].

**Table 2:** Summary of Behavioural Observations.

Dist.	Pairs	Trials	A	S	B	L	C	R	Proximity score
5m	7	54	20	8	26	7	15	32	3.12
10m	5	48	20	10	18	4	12	32	3.37
*p*			*0.48*	*0.13*	*0.93*	*0.83*	*0.78*	*0.22*	*0.41*

This table provides the data from the two sets of experiments, representing releases at initial separations of 5m and 10m. Shown are the number of *Pairs*, the number of *Trials* for each experimental condition, the respective scores for the reference-bird’s preference to fly either above (*A*), at the same height (*S*) or below (*B*) the other bird, the scores for the reference-bird preference to move to the left (*L*), fly along the center (*C*) or fly to the right (*R*), as well as the average proximity score. Finally, the table also provides p-values for the two-sample T-test comparison of all scored behaviours for the two different release distances.

**Fig 2:**
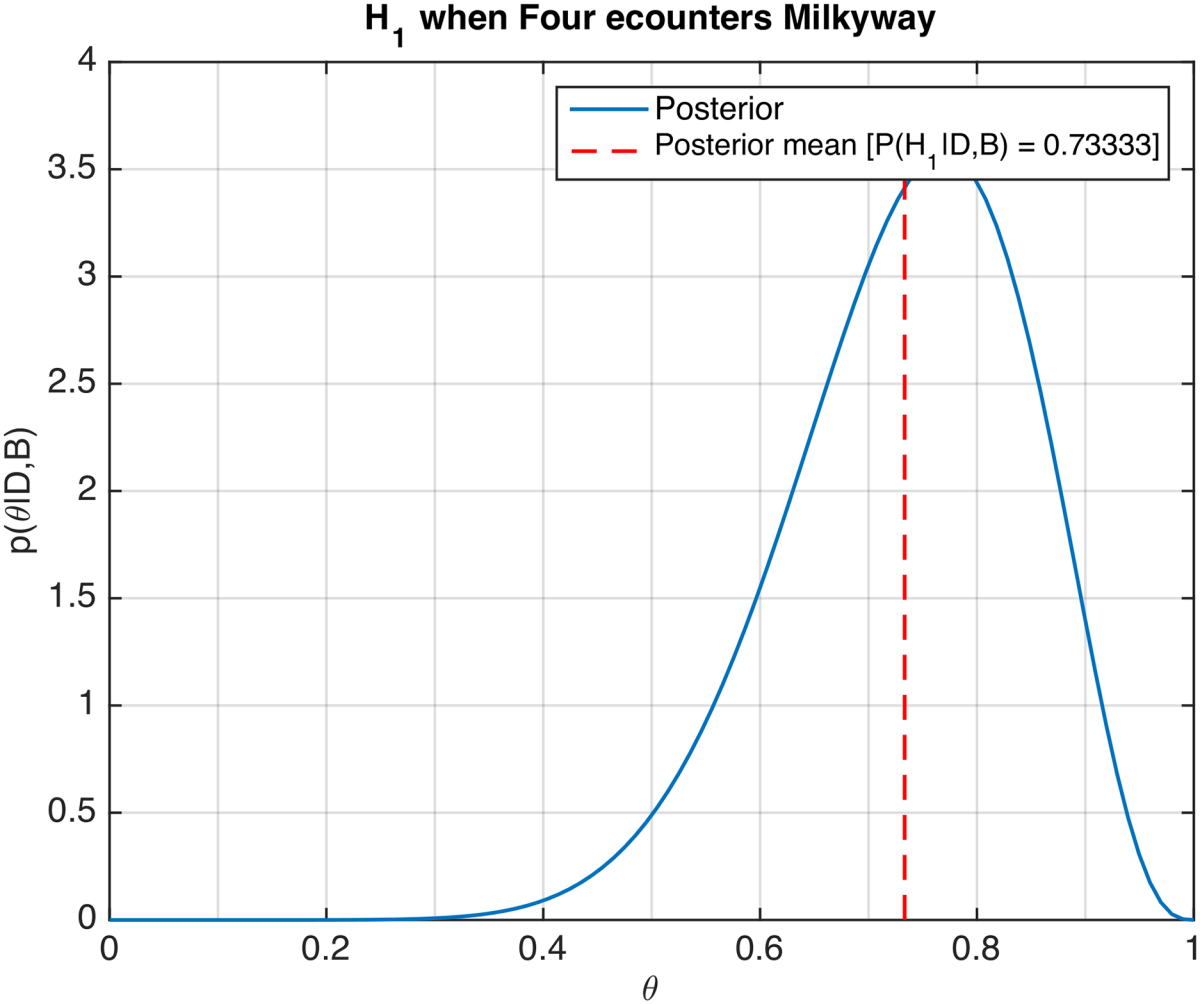
Posterior distribution of veering probability. Figure shows the computed probability distribution *p*(*θ*|*D*, *B*_1*jk*_) of the reference bird ‘Four’ veering to the right when encountering ‘Milkyway’. The red dashed line indicates the mean of the posterior distribution, i.e. the predicted probability $P(H_{1}^{jk}|D)$.

As a result of this initial analysis we will, for the remainder of the study, pool the two data sets and consider them as one. A comprehensive summary of all of the results, for each pair of birds and each reference bird is given in S1A Table. A generally observed behavior, immediately evident from the data in Table 1, was an overall tendency for the birds to pass each other on the left—i.e. each bird moved to its right to avoid a collision. Fig 2 shows an example of the posterior distribution and the predictive probabilities for Hypothesis 1, based on tests conducted on the reference bird called ‘Four’ when encountering the bird called ‘Milkyway’. From these results, we conjecture, that for this pair, ‘Four’ shows a preference to fly to the right of ‘Milkyway’. These computations are based on 20 head-on encounters. Details of the analysis are given in S1 Supplementary Methods.

A detailed analysis supported these initial observations (See Table 3), with 6 of the 7 pairs displaying probabilities in favor of the theory that birds pass each other on the left, by moving to the right (overall predicted probability *P*(*H*_1_|*D*, *B*_1*jk*_) = 0.84).

**Table 3:** Preference of the reference bird to move to the left or the right.

Bird 1	Bird 2	Trials	L	C	R	*P*(*H*_1_|*D*, *B*_1*jk*_)
Blackhole	Nemo	9	7	1	1	0.20
Drongo	Four	3	0	1	2	0.75
Drongo	Three	19	0	4	15	0.94
One	Two	19	0	0	19	0.95
Four	Milkyway	20	3	7	10	0.73
Nemo	Three	19	0	8	11	0.92
Rama	Titan	13	1	6	6	0.78
**Sum**		**102**	**11**	**27**	**64**	**Avg. 0.84**

Summary of the observations and respective scores for each of the 7 Pairs of Birds (*Bird 1 and Bird 2*), the number of *Trials* for each pairs, the respective scores for the reference bird’s preference to move to the left *L*, fly along the center *C* or fly to the right *R*, and the predicted probability *P*(*H*_1_|*D*, *B*_1*jk*_) for the preference of the reference bird in a given pair to move to the right.

These results are interesting because we know, from general observations as well as other studies (unpublished data) that budgerigars also show a tendency to fly to the right of the mid-line when flying alone. However, other studies in budgerigars have shown that the biases displayed by these birds vary strongly from individual to individual and are also highly dependent on the task at hand [11, 12]. So far, in the context of short range guidance maneuvers, a consistent bias across all individuals has been observed only in a task in which the birds are required to choose between two landing perches, one to the left and the other to the right of their flight direction. Here the birds display a significant preference for the right-hand perch, at the level of the population [11].

Collision avoidance studies in bees suggest that bees, unlike budgerigars, show a bias to move towards the left to avoid collisions (unpublished data). In the context of collision avoidance in head-on encounters, the need for a bias that is consistent across all individuals is obviously of paramount importance—random biases across individuals would not be favorable, as they would lead to collisions in half of the encounters, on average. The presence of a bias is in line with general theories about biases in behaviors arising as a consequence of relegating the control of different tasks to different hemispheres of the brain, where in the case of the budgerigar the left eye and right hemisphere could be responsible for collision avoidance, allowing the right eye and left hemisphere to focus on other tasks such as flight maintenance and speed control [13].

**Fig 3:**
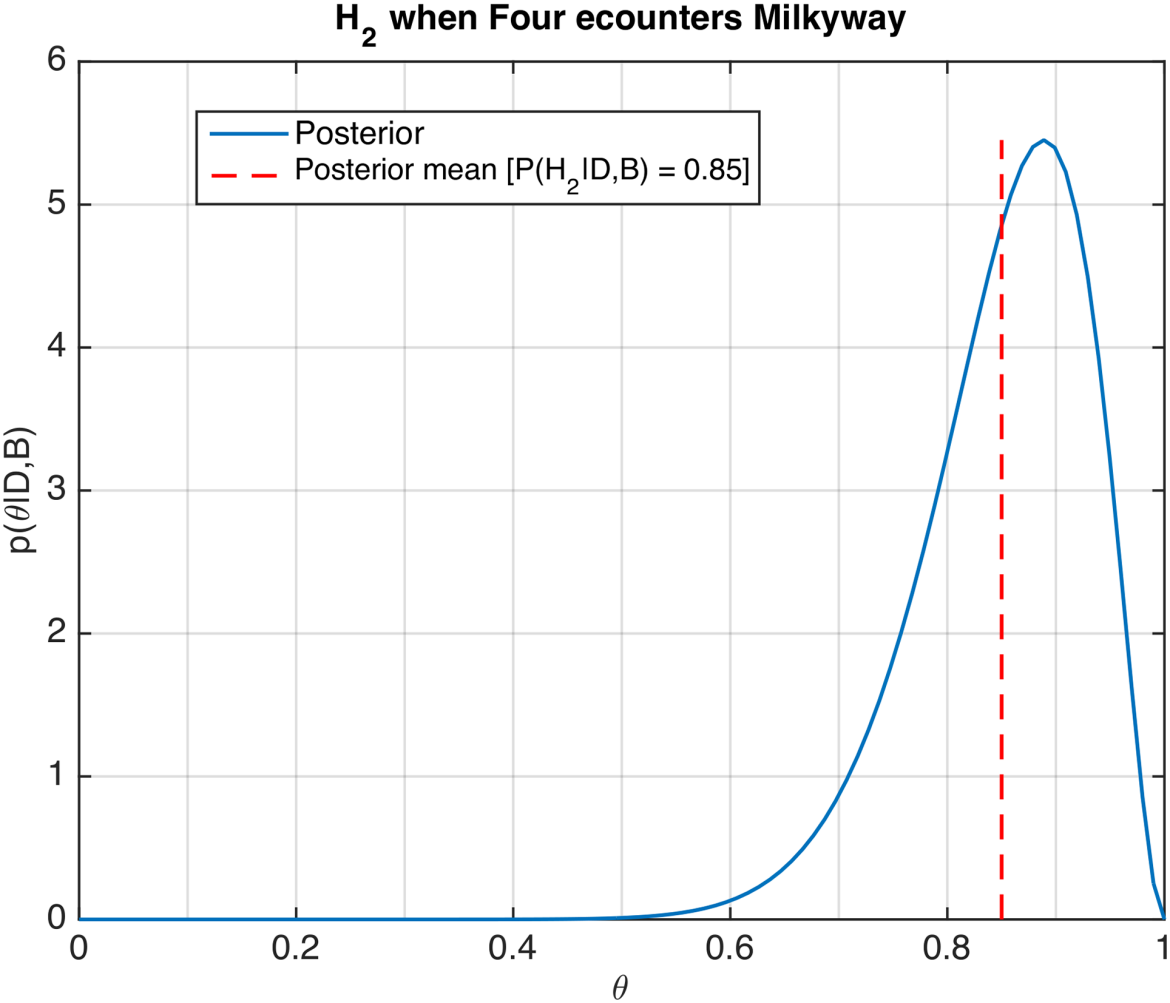
Posterior distribution of probability to change altitude. Figure shows the computed probability distribution *p*(*θ*|*D*, *B*_2*jk*_) of the reference bird ‘Four’ switching to a lower altitude when encountering ‘Milkyway’. The red dashed line indicates the mean of the posterior distribution, i.e. the predicted probability $P(H_{2}^{jk}|D)$.

Another observation was that birds would rarely fly at the same height, allowing passage to occur at different heights, thus decreasing the risk of collision during passage even further. This raised the question whether individual birds had a specific preference to fly higher or lower. Fig 3 shows an example of the posterior distribution and the predictive probability for Hypothesis 2, based on tests conducted on the reference bird called ‘Four’ when encountering the bird called ‘Milkyway’. From these results, we conjecture that for this pair, ‘Four’ shows a preference to fly below ‘Milkyway’. These computations are based on 20 head-on encounters. Details of the computations are given in S1 Supplementary Methods.

In an analysis similar to that described above we found clear evidence for the hypothesis that individual birds displayed a preference to pass the other bird by either flying above or below the other bird (averaged predicted probability in favor of the hypothesis was *P*(*H*_2_|*D*, *B*_2*jk*_) = 0.79, See Table 4).

**Table 4:** Preference of the reference bird to fly above or below the other bird.

Bird 1	Bird 2	Trials	A	S	B	*P*(*H*_2_|*D*, *B*_2*jk*_)
Blackhole	Nemo	9	5	2	2	0.67
Drongo	Four	3	2	1	0	0.75
Drongo	Three	19	16	2	1	0.89
One	Two	19	3	5	11	0.75
Four	Milkyway	20	2	2	16	0.85
Nemo	Three	19	0	3	16	0.94
Rama	Titan	13	3	3	7	0.67
**Sum**		**102**	**31**	**18**	**53**	**Avg. 0.79**

Summary of the observations and respective scores for each of the 7 Pairs of Birds (*Bird 1 and Bird 2*), the number of *Trials* for each pair, the respective scores for the reference bird’s preference to fly above *A*, at the same height as the other bird *S* or below *B* the other bird, and the predicted probability *P*(*H*_2_|*D*, *B*_1*jk*_) for the reference bird’s preference in a given pair to fly above or below the other bird.

While it is relatively straightforward to ensure that all birds maintain a consistent left or right bias (as all birds only need to have the same bias), solving the issue of which bird has to fly higher and which bird has to fly lower is rather difficult.

One possible solution could have been that birds flying along a specific compass direction would always fly on top of the other bird, and birds flying in the opposite direction would always fly below. This should be feasible, at least in principle, as many birds are likely to possess a magnetic compass [14]. Thus we can define an additional hypothesis:

$$H_{3}=\left\{\text{flight}\;\text{direction}\;\text{affects}\;\text{the}\;\text{avoidance}\;\text{behaviour.}\right\}$$ (1)

However, all budgerigars were flown in both directions in the experiments, and the behavior of each individual was independent of the flight direction. The predicted probability for a preference to fly on top when flying eastbound—The tunnel was oriented roughly in the east-west direction—was *P*(*H*_3_|*D*) = 0.52. Therefore we can reject the hypothesis that the direction of flight in the tunnel influences avoidance behaviours.

This leaves two other possible solutions. One possible solution is that individual budgerigars have a preference to fly at specific heights. Another possible solution is that when two budgerigars fly toward each other, their positions in the group hierarchy determine which individual moves up and which moves down. While the latter cannot be excluded based on the current data, we know from observational experience that individual birds do prefer to fly at specific heights even when flying alone in the tunnel. This said, further studies are needed to confirm whether group hierarchy influences collision-avoidance behaviours.

The finding of the right-veering bias across most of the individuals in our tested population is consistent with game-theoretical analyses that point out the benefits of a population—level bias, and predict its presence in animals living in groups (social animals) [15]. Budgerigars are clearly social animals, moving in flocks that can contain up to a hundred birds, and occasionally a few thousand [16].

## Conclusion

We have designed and conducted experiments to investigate how birds avoid mid-air collisions in head-on encounters. We have posed hypotheses about avoidance behaviours and used the experimental data to assess their truth or falsity.

Our analysis reveals that birds exhibit a preference to veer to the right, and maintain a preferred altitude to avoid collisions in head-on encounters. We also find that collision avoidance behavior does not depend upon the direction of flight, thus ruling out the possible involvement of an internal compass.

These strategies suggest simple rules by which collisions can be avoided in head-on encounters by two agents, be they animals or machines. Firstly, each agent needs to have a consistent preference to move to one side. This can either be to the left or the right, but it has to be consistent across all agents. Secondly, if height maneuvers are also involved, they need to be established either a priori, or implemented in real time in an impromptu fashion. This can be achieved in various ways. One approach would be to arrange all flying agents in the sky in a hierarchical order by assigning a number to each agent to decide which agent moves higher and which moves lower during a head-on encounter. (For example, the rule could be that higher ranked agent loses height while the lower ranked agent gains height.). A universal hierarchy is, of course, not easy to implement and enforce, and there is a need for the two agents to exchange information about their hierarchies before the requisite height changes can be orchestrated. This communication will require a finite time and increase the risk of a collision. Another strategy would be to arrange for each agent to move randomly up or down, independently of the other agent. This will reduce the risk of collision in half of the encounters, on average, but if it is combined with the strategy of veering in the horizontal plane, it will serve to increase the separation in half of the encounters. However, a strategy in which the increase or decrease of height is determined by the direction of flight would be a robust and reliable solution that would not require the establishment of a hierarchy or communication between the agents. While we cannot say how birds solve the problem of switching to different altitudes, our findings suggest a number of simple strategies that can be implemented in autopilot systems and unmanned aerial vehicles to prevent head-on collisions.

## Supporting Information

S1 Supplementary Methods Computation of Predictive Probabilities. (PDF)

S1 Table Detailed Behavioral Observations. (PDF)
