# Supplementary material for: Strategies for Pre-Emptive Mid-Air Collision Avoidance in Budgerigars
Source: PLoS One. 2016 Sep 28;11(9):e0162435. doi: 10.1371/journal.pone.0162435 (PMC5040264; doi:10.1371/journal.pone.0162435)
Supplement: S1 Supplementary Methods — (PDF) [file pone.0162435.s001.pdf]

S1 Supplementary Methods - Computation of Predictive Probabilities

Ingo Schiffner,Tristan Perez, Mandyam V. Srinivasan

Inference

If we consider the bird j and we conduct a series of N^{jk} experiments when encountering the bird k, then for each experiment, we can evaluate whether the behaviour stated by the hypothesis H_i^{jk} $(i = 1, 2)$ has been observed:

- $H_1^{jk} = \{$the reference bird j has a preference to veer towards the right when encountering bird k $\}$;
- $H_2^{jk} = \{$the reference bird j has a preference to increase its altitude when encountering bird k $\}$.

This will lead to a sequence of binary data (1):

$$D = \{d_1, d_2, \ldots, d_{N^{jk}}\}, \tag{1}$$

where

$$d_l = \begin{cases} 1 & \text{if the behaviour behaviour stated by} H_i^{jk} \text{is observed in the experiment } l, \\ 0 & \text{otherwise.} \end{cases} \tag{2}$$

If the N^{jk} experiments are independent, then the probability of obtaining a particular dataset of the form (1) is given by

$$P(D|\theta, B_{ijk}) = \prod_{l=1}^{N^{jk}} (\theta)^{d_l} (1-\theta)^{1-d_l} = (\theta)^{R_{ikj}} (1-\theta)^{N^{jk} - R_{ijk}}, \tag{3}$$

where B_{ijk} represents the background information that states we are considering the hypothesis i $(i = 1, 2)$ for the bird j encountering the bird k. The parameter $0 \leq \theta \leq 1$ represents the probability of observing the behaviour associated with H_i^{jk} in one experiment, $R_{ijk} = \sum_l d_l$ is the number of favourable observations of behaviour associated with H_i^{jk} in the N^{jk} experiments.

Expression (3) can be used as a likelihood function to infer the value of the parameter θ. If the initial uncertainty of this parameter is described using a prior distribution $p(\theta|B_{ijk})$, then we can update a prior distribution for the parameter to a posterior distribution using the Bayes's Theorem:

$$p(\theta|D, B_{ijk}) = \frac{p(D|\theta, B_{ijk})\, p(\theta|B_{ijk})}{p(D|B_{ijk})}. \tag{4}$$

Prediction

Having computed the posterior distribution $p(\theta|D, B_{ijk})$ for the parameter θ using (4), the question that arises is *what is the probability of obtaining a certain number of behaviours H_i^{jk} in a number of future experiments?*

If we knew the true value of θ, then the probability of having Z successes in M experiments is given by the Binomial distribution:

$$p(Z|\theta, M) = \binom{M}{Z} \theta^Z (1-\theta)^{M-Z}. \tag{5}$$

Through the inference process described in the previous section, we only know its posterior (4) and not the actual value of θ. We could pick a point estimate, say the mode or the median, and substitute it in (5), but this would ignore the

uncertainty about θ that the posterior encodes, and we would throwing away information. To make a better use all of the information available, the predicted probability of Z given the data D can be computed by marginalisation:

$$p(Z|D) = \int_0^1 p(Z,\theta|D)\,d\theta, \tag{6}$$

$$= \int_0^1 p(Z|\theta)\,p(\theta|D)\,d\theta_i^j. \tag{7}$$

By doing this integration, we take into account the uncertainty about θ.

The required probabilities $P(H_i^{jk}|D)$ reported in the paper can be taken, by choice, as the predicted probabilities of observing the associated behaviour in one future experiment. These can be computed by taking Z=1 and M=1 in (5), in which case (6) reduces to the mean of the posterior:

$$P(H_i^{jk}|D) = \int_0^1 \theta\,p(\theta|D)\,d\theta. \tag{8}$$

For the particular problem of interest in this paper, we advocate the use of a uniform prior distribution $p(\theta|B_{ijk})$ in (4). The adoption of a uniform prior distribution, or Bayes-Laplace prior, for the parameter θ reflects our unassuming attitude towards the exhibited behaviours of the bird j—that is, θ could take any value in the range from 0 to 1. This follows from the Maximum Entropy Principle given that there may be no testable information [1–3]. Further details as to why this a compelling choice of a prior for the type of experiments being considered in this paper are discussed in [4]. If we adopt a uniform distribution for the prior $p(\theta|B_{ijk})$, then the posterior (4) takes the following form ([2] p 165):

$$p(\theta|D, B_{ijk}) = \frac{(N+1)!}{(R+1)!\,(N-R)!}\,(\theta)^R(1-\theta)^{N-R}, \tag{9}$$

and the predictive probabilities (8) become:

$$P(H_i^{jk}|D) = \frac{R+1}{N+2}, \tag{10}$$

where for simplicity of notation $R \equiv R_{ijk}$ and $N \equiv N^{jk}$.

References

1. Jaynes ET. Where Do We Stand on Maximum Entropy? (1978). In: Rosenkrantz RD, editor. E. T. Jaynes: Papers on Probability, Statistics and Statistical Physics. vol. 158 of Synthese Library. Springer Netherlands; 1989. p. 210–314.

2. Jaynes ET. Probability Theory, The Logic of Science. Cambridge University Press; 2003.

3. Gregory P. Bayesian Logical Data Analysis. Cambridge University Press; 2005.

4. Geisser S. On Prior Distributions for Binary Trials. The American Statistician. 1984;38(4):244–247.
