# Supplementary material for: Strategies for Pre-Emptive Mid-Air Collision Avoidance in Budgerigars
Source: PLoS One. 2016 Sep 28;11(9):e0162435. doi: 10.1371/journal.pone.0162435 (PMC5040264; doi:10.1371/journal.pone.0162435)
Supplement: S1 Table — (PDF) [file pone.0162435.s002.pdf]

S1 Table - Detailed Behavioral Observations

Ingo Schiffner,Tristan Perez, Mandyam V. Srinivasan

Table A. Detailed Behavioural Observations.

Date	Bird 1	Bird 2	Pair	Trials	Dist.(m)	Above	Same	Below	Left	Center	Right	Proximity score
28/01/14	Blackhole	Nemo	1	5	10	3	1	1	4	0	1	2.60
28/01/14	Nemo	Blackhole	1	4	10	1	1	2	3	1	0	2.75
28/01/14	Drongo	Four	2	1	10	0	1	0	0	1	0	1.00
28/01/14	Four	Drongo	2	2	10	0	0	2	0	0	2	2.50
4/02/14	Drongo	Three	3	5	10	4	0	1	0	3	2	3.40
4/02/14	Three	Drongo	3	5	10	0	1	4	0	0	5	3.40
28/01/14	One	Two	4	4	10	0	1	3	0	0	4	3.75
28/01/14	Two	One	4	5	10	1	1	3	0	0	5	3.20
4/02/14	Four	Milkyway	5	5	10	0	1	4	0	2	3	3.40
4/02/14	Milkyway	Four	5	5	10	5	0	0	0	2	3	3.40
4/02/14	Nemo	Three	6	5	10	0	1	4	0	0	5	3.20
4/02/14	Three	Nemo	6	5	10	5	0	0	0	3	2	3.60
4/02/14	Rama	Titan	7	2	10	0	0	2	0	2	0	3.50
4/02/14	Titan	Rama	7	1	10	1	0	0	0	1	0	4.00
7/02/14	Drongo	Three	3	4	5	3	1	0	0	1	3	4.00
7/02/14	Three	Drongo	3	5	5	0	0	5	0	0	5	3.80
7/02/14	One	Two	4	5	5	0	2	3	0	0	5	4.00
7/02/14	Two	One	4	5	5	4	1	0	0	0	5	3.60
10/02/14	Four	Milkyway	5	5	5	2	0	3	2	2	1	2.20
10/02/14	Milkyway	Four	5	5	5	4	1	0	1	1	3	3.60
10/02/14	Nemo	Three	6	4	5	0	2	2	0	2	2	2.25
10/02/14	Three	Nemo	6	5	5	5	0	0	0	3	2	3.20
10/02/14	Rama	Titan	7	5	5	1	1	3	1	1	3	3.60
10/02/14	Ritan	Rama	7	5	5	1	2	2	0	2	3	3.40

This table provides data for each individual bird for each pair and the respective number of Trials during which the birds flew in this specific configuration from the two sets of experiments (releases at 5m and 10m), Shown are also the respective scores for the reference-bird's preference to fly either above, at the same height or below the other bird, the scores for the reference-bird preference to move to the left, fly along the center or fly to the right, as well as the average proximity score.
